# The SUMO E3 ligase, *AtSIZ1*, regulates flowering by controlling a salicylic acid-mediated floral promotion pathway and through affects on *FLC* chromatin structure

**DOI:** 10.1111/j.1365-313X.2007.03359.x

**Published:** 2008-02

**Authors:** Jing Bo Jin, Yin Hua Jin, Jiyoung Lee, Kenji Miura, Chan Yul Yoo, Woe-Yeon Kim, Michael Van Oosten, Youbong Hyun, David E Somers, Ilha Lee, Dae-Jin Yun, Ray A Bressan, Paul M Hasegawa

**Affiliations:** 1 Department of Horticulture and Landscape Architecture, Purdue University West Lafayette, IN 47907 2010, USA; 2 Division of Applied Life Science (BK21 program), and Environmental Biotechnology National Core Research Center, Gyeongsang National University Jinju 660 701, Korea; 3 Department of Plant Cellular and Molecular Biology, Plant Biotechnology Center, Ohio State University Columbus, OH 43210, USA; 4 Department of Biological Sciences, Seoul National University Seoul 151 742, Korea

**Keywords:** *SIZ1*, SA, flowering, SUMO, *FLD*, *FLC*

## Abstract

Loss-of-function *siz1* mutations caused early flowering under short days. *siz1* plants have elevated salicylic acid (SA) levels, which are restored to wild-type levels by expressing *nahG*, bacterial salicylate hydroxylase. The early flowering of *siz1* was suppressed by expressing *nahG*, indicating that *SIZ1* represses the transition to flowering mainly through suppressing SA-dependent floral promotion signaling under short days. Previous results have shown that exogenous SA treatment does not suppress late flowering of autonomous pathway mutants. However, the *siz1* mutation accelerated flowering time of an autonomous pathway mutant, *luminidependens*, by reducing the expression of *FLOWERING LOCUS C* (*FLC*), a floral repressor. This result suggests that *SIZ1* promotes *FLC* expression, possibly through an SA-independent pathway. Evidence indicates that *SIZ1* is required for the full activation of *FLC* expression in the late-flowering *FRIGIDA* background. Interestingly, increased *FLC* expression and late flowering of an autonomous pathway mutant, *flowering locus d* (*fld*), was not suppressed by *siz1*, suggesting that *SIZ1* promotes *FLC* expression by repressing FLD. Consistent with this, SIZ1 facilitates sumoylation of FLD that can be suppressed by mutations in three predicted sumoylation motifs in FLD (i.e. FLDK3R). Furthermore, expression of *FLDK3R* in *fld* protoplasts strongly reduced *FLC* transcription compared with expression of *FLD*, and this affect was linked to reduced acetylation of histone 4 in *FLC* chromatin. Taken together, the results suggest that *SIZ1* is a floral repressor that not only represses the SA-dependent pathway, but also promotes *FLC* expression by repressing FLD activity through sumoylation, which is required for full *FLC* expression in a *FRIGIDA* background.

## Introduction

Sumoylation is a post-translational regulatory process that conjugates small ubiquitin modifier peptides (SUMO) to protein substrates (Matunis *et al.*, 1996; Mahajan *et al.*, 1997). Like ubiquitination, SUMO attachment to a target substrate involves a series of steps referred to as activation (E1), conjugation (E2) and ligation (E3) (Seeler and Dejean, 2003; Johnson, 2004). SUMO E3 ligases of the PIAS/SIZ family facilitate SUMO conjugation to lysine (K) residues in the SUMO consensus motif, ΨKXE/D (Ψ, a large hydrophobic residue; K, the acceptor lysine; X, any amino acid; E/D, glutamate or aspartate), located in protein substrates (Schmidt and Muller, 2003). SUMO modification of target proteins in yeast and metazoans has been implicated in the regulation of innate immunity, cell-cycle progression and mitosis, DNA repair, chromatin stability, nucleocytoplasmic trafficking, subnuclear targeting, ubiquitination antagonism and transcriptional regulation (Johnson, 2004; Gill, 2005). Sumoylation in plants is reported to be involved in biotic and abiotic stress responses, flowering and development (Chosed *et al.*, 2006; Downes and Vierstra, 2005; Kurepa *et al.*, 2003; Lee *et al.*, 2007; Miura *et al.*, 2005, 2007; Novatchkova *et al.*, 2004; Yoo *et al.*, 2006). The Arabidopsis PIAS-type SUMO E3 ligase, *AtSIZ1*, facilitates SUMO modification of transcription factors, PHR1 and ICE1, which regulate phosphate-starvation signaling and low-temperature response, respectively (Miura *et al.*, 2005, 2007). A SUMO protease, AtESD4 (EARLY SHORT DAY FLOWERING4), and its interacting protein NUA (NUCLEAR PORE ANCHOR) negatively regulate transition to flowering, suggesting that SUMO homeostasis is important for flowering time regulation (Murtas *et al.*, 2003; Reeves *et al.*, 2002; Xu *et al.*, 2007).

Flowering is the result of a plant developmental process that controls the transition from vegetative maturity to the reproductive stage (Baurle and Dean, 2006). Floral transition is regulated by day length, light quality and temperature, and this responsive capacity is thought to optimize the environmental fitness of plants (Ballare, 1999; Corbesier *et al.*, 1996; Michaels and Amasino, 2001). The vegetative to floral transition of Arabidopsis, and other rosette-type plants, is characterized by the rapid proliferation of an extended floral shoot that is the result of internodal expansion (Blazquez *et al.*, 1997). In Arabidopsis, signal regulatory cascades, such as the photoperiodic- (or long-day), vernalization, autonomous and gibberellin (GA)-dependent pathways, control floral transition (He and Amasino, 2005).

Photoperiodic-pathway genes promote transition to flowering in response to a long-day photoperiod (Koornneef *et al.*, 1991). Mutations to photoperiodic-pathway genes, such as *GIGANTEA* (*GI*), *CONSTANS* (*CO*) and *FLOWERING LOCUS T* (*FT*), cause significantly delayed flowering under long days (Koornneef *et al.*, 1991). Rhythmic expression of *CO* transcript is regulated by circadian clock oscillators [e.g. *CIRCADIAN CLOCK-ASSOCIATED PROTEIN 1* (*CCA1*)] (Green and Tobin, 1999) and clock- and light-regulated genes (i.e. *GI*) (Fowler *et al.*, 1999). Under long days (LD) CO protein accumulates to levels that promote floral transition, mainly through activation of *FLOWERING LOCUS T* (*FT*) expression. FT in turn activates expression of *SUPPRESSOR OF OVEREXPRESSION OF CONSTANS 1* (*SOC1*) and floral identity genes such as *APETALA1* (*AP1*) (Yoo *et al.*, 2005; Wigge *et al.*, 2005).

In contrast to the photoperiodic pathway, which directly activates the floral transition, the vernalization and autonomous pathways indirectly promote transition to flowering through repression of the central floral repressor, *FLOWERING LOCUS C* (*FLC*) (Michaels and Amasino, 1999). *FLC* encodes a MADS box-containing transcription factor that antagonizes floral transition facilitated by the photoperiodic pathways, by repressing *FT* and *SOC1* expression (Michaels and Amasino, 1999; Searle *et al.*, 2006). The presence of an active allele of *FRIGIDA* (*FRI*, an activator of *FLC*) in winter-annual ecotypes causes increased expression of *FLC* and delayed flowering, which is reversed by lesions in *FLC* or by vernalization treatment (Michaels and Amasino, 1999; Sheldon *et al.*, 1999). To date, eight autonomous pathway genes have been identified from screening for late-flowering mutants that are responsive to daylength and vernalization treatment (Koornneef *et al.*, 1991; Schmitz and Amasino, 2007). Among the autonomous pathway genes, *FLOWERING LOCUS D* (*FLD*), a plant ortholog of the human protein *KIAA0601*/*LYSINE-SPECIFIC HISTONE DEMETHYLASE1* (*LSD1*), represses *FLC* expression by facilitating deacetylation of histone H4 in *FLC* chromatin, but how this process is regulated remains to be elucidated (He *et al.*, 2003; Shi *et al.*, 2004).

In addition to light and temperatures, the plant hormones GA and salicylic acid (SA) are implicated in the regulation of floral transition (Cleland and Ben-Tal, 1982; Wilson *et al.*, 1992). GA promotes flowering through the activation of *SOC1* and *LEAFY* expression, and is considered to be involved in the main floral-inducing cascade under short days (SD) (Blazquez *et al.*, 1998; Langridge, 1957; Moon *et al.*, 2004). Exogenous SA treatment or UV-C light stress, which induces accumulation of SA, accelerates the transition to flowering (Martinez *et al.*, 2004). SA-deficient *nahG*-expressing plants, or *eds5*/*sid1* and *sid2* mutants, exhibit a delayed flowering phenotype that is evident under SD (Martinez *et al.*, 2004). SA control of the transition to flowering appears to be complex, and the extent of its role remains to be elucidated (Martinez *et al.*, 2004).

Although sumoylation/desumoylation has been implicated in flowering-time regulation, target proteins that are involved in flowering remain unknown (Murtas *et al.*, 2003; Xu *et al.*, 2007). In this report, evidence indicates that FLD is a sumoylation target for SIZ1. SUMO conjugation to FLD inhibits its activity to repress *FLC* expression, which is required for full activation of *FLC* expression in a *FRI* background. Our results also demonstrate that the early flowering of *siz1* in SD is mainly the result of an elevated SA level. This and other results (Lee *et al.*, 2007) indicate that sumoylation has an important role in the regulation of SA accumulation, although the sumoylation targets involved in SA accumulation are unidentified.

## Results

### The SUMO E3 ligase SIZ1 regulates flowering time

**Figure1:**
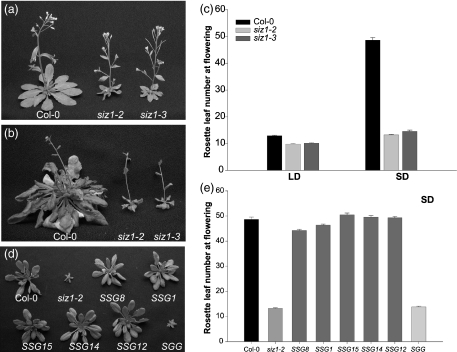
*SIZ1* represses transition to flowering. (a) Wild-type (Col-0) and *siz1* (*siz1*-*2* and *siz1*-*3*) plants were grown under long days (LD) for 32 days. (b) Wild-type (Col-0) and *siz1* (*siz1*-*2* and *siz1*-*3*) plants were grown under short days (SD) for 113 and 50 days, respectively. (c) The number of rosette leaves at flowering of wild-type and *siz1* plants. Plants were grown under LD or SD. (d) Phenotypic comparison of plants: wild-type (Col-0), *siz1*-*2* and transgenic *Pro*_*SIZ1*_*:SIZ1:GFP* expressing *siz1*-*2* (*SSG1,8,12,14,15*) and *Pro*_*SIZ1*_*:GUS:GFP* expressing *siz1-2* (*SGG*) plants. (e) The number of rosette leaves at the flowering of plants, as in (d), which were grown under SD. Data illustrated in (c) and (e) are means ± SE of 15–20 plants per analysis.

Flowering time of Col-0 wild-type and *siz1* (*siz1*-*2* and *siz1*-*3*) loss-of-function mutant plants (Miura *et al.*, 2005) under LD (16-h light and 8-h dark) or SD (8-h light and 16-h dark) conditions (Lee *et al.*, 1994a) indicated that *SIZ1* negatively regulates the transition to flowering (Figure 1a–c). Flowering time of *siz1* plants relative to wild type was slightly earlier under LD, and was substantially earlier under SD (Figure 1a–c). Rosette leaf numbers at flowering under LD and SD were 10 and 13, respectively, for *siz1* plants, and were 13 and 49, respectively, for wild type (Figure 1c). Thus, the floral transition of *siz1* plants is much less photoperiodic responsive than that of wild type.

The role of *SIZ1* in flowering-time regulation was confirmed by genetic complementation of the *siz1* mutation with the wild-type *SIZ1* allele. Expression of *Pro*_*SIZ1*_*:SIZ1:GFP* (SSGs) in *siz1*-*2* plants suppressed the dwarf and early flowering phenotypes of plants from multiple, independent transformed lines (Figure 1d,e). Expression of *Pro*_*SIZ1*_*:GUS:GFP* (SGG) in *siz1*-*2* failed to complement these *siz1*-*2* phenotypes in the same experiments (Figure 1d,e).

### SIZ1 regulates flowering, independent of the photoperiodic- and GA-dependent pathways

Mutations in circadian oscillator genes cause a short-period phenotype, which results in early flowering under SD (Mizoguchi *et al.*, 2002). To test whether *siz1* affects the circadian clock, the rhythmic expression patterns of *CCA1* and *COLD CIRCADIAN RHYTHM RNA BINDING 2* (*CCR2*) were determined (Green and Tobin, 1999; Kreps and Simon, 1997). The diurnal rhythmic expression of *CCA1* and *CCR2::LUCIFERASE* (*CCR2::LUC*) was not altered by *siz1* mutations, suggesting that *SIZ1* does not regulate the circadian clock (Figures S1A,B, respectively).

**Figure2:**
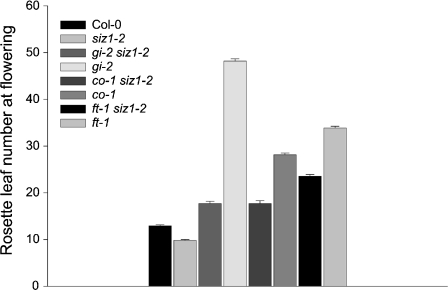
*siz1* partially suppresses late-flowering photoperiodic-pathway mutants. The flowering times of wild-type (Col-0), *siz1*-*2*, *gi*-*2* *siz1*-*2*, *gi*-*2*, *co*-*1 siz1*-*2*, *co*-*1*, *ft*-*1 siz1*-*2* and *ft*-*1* plants were estimated under long days (LD). Data are means ± SE of 15–20 plants per analysis.

To determine if there is an interaction between *SIZ1* and the photoperiodic-pathway genes *GI*, *CO* and *FT*, the double mutants *gi*-*2 siz1*-*2*, *co*-*1 siz1*-*2* and *ft*-*1 siz1*-*2* were produced. Flowering times of the double mutants were intermediate to that of *siz1*-*2* and the corresponding late-flowering parental mutant plants under LD, suggesting that *SIZ1* may function independently of the photoperiodic pathway (Figure 2). Consistent with this hypothesis, expression patterns of *GI*, *CO* and *FT* were not altered by *siz1* (data not shown). To determine whether *siz1* mutations affect the GA-dependent floral promotion pathway, exogenous GA was applied to wild-type, *siz1*-*2* and *siz1*-*3* plants, and flowering times were determined under LD and SD (Figure S2). Exogenous GA treatment accelerated the flowering time of both wild-type and *siz1* plants to an equivalent extent (Figure S2), indicating that the GA floral promotion pathway is not impaired in *siz1* plants.

### SIZ1 regulates flowering mainly through an SA-dependent pathway in the Columbia background

**Figure3:**
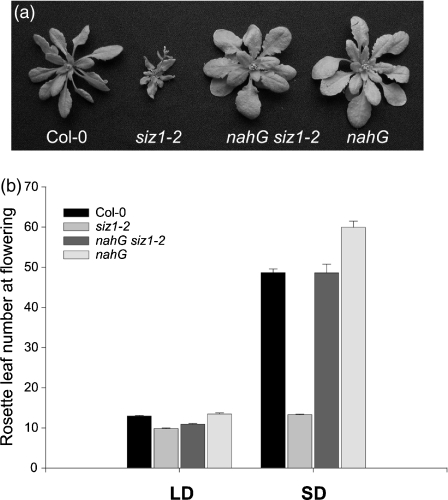
Early flowering of *siz1* under short days (SD) is mainly caused by elevated salicylic acid (SA) levels. (a) Wild-type (Col-0), *siz1*-*2*, *nahG* *siz1*-*2* and *nahG* plants were grown under long days (LD). (b) Number of rosette leaves at flowering of wild-type, *siz1*-*2*, *nahG* *siz1*-*2* and *nahG* plants. Plants were grown under LD or short days (SD). Data are means ± SE of 15–20 plants per analysis.

Compared with wild-type Col-0 plants, *siz1* plants accumulate higher levels of SA, which causes increased plant innate immunity (Lee *et al.*, 2007). The bacterial gene *nahG* encodes a protein that rapidly and efficiently converts SA to inactive catechol *in planta* (Delaney *et al.*, 1994). To elucidate SA effects on the *siz1* early flowering phenotype, flowering times of wild-type, *siz1*-*2*, *nahG* *siz1*-*2* and *nahG* plants were compared (Figure 3a,b). In a previous report, we have shown that *nahG* and *nahG* *siz1*-*2* plants accumulate SA at levels similar to wild-type plants (Lee *et al.*, 2007). *nahG siz1*-*2* plants also exhibit nearly normal leaf morphology and rosette plant size (Figure 3a; Lee *et al.*, 2007). Flowering time of *nahG* *siz1*-*2* plants was similar to that of *siz1*-*2* plants under LD, but similar to *nahG* and wild-type plants under SD (Figure 3b). These results indicate that in the Columbia genetic background, the early flowering phenotype of *siz1* is mainly dependent on an SA-dependent pathway under SD, but not under LD.

### The siz1 early flowering phenotype is in part linked to reduced MAF4 expression and elevated SOC1 expression

**Figure4:**
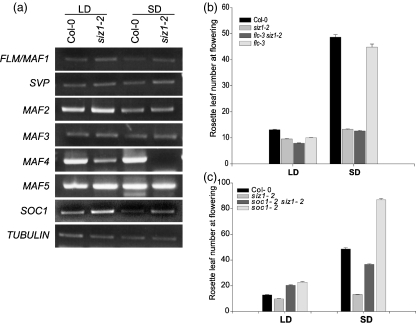
*SIZ1* represses *SOC1* expression but activates *MAF4* expression. (a) *FLM/MAF1*, *SVP*, *MAF2*, *MAF3*, *MAF4*, *MAF5* and *SOC1* mRNA levels in wild-type (Col-0) and *siz1*-*2* plants were determined by RT-PCR. RNA was isolated from 14-day-old seedlings grown under long days (LD) or short days (SD). *TUBULIN* was used as a control for loading. (b, c) Flowering time of wild-type (Col-0), *siz1*-*2*, *flc*-*3* *siz1*-*2*, *flc*-*3*, *soc1*-*2* *siz1*-*2* and *soc1*-*2* plants were estimated under LD and SD. Data are means ± SE of 15–20 plants per analysis.

**Figure5:**
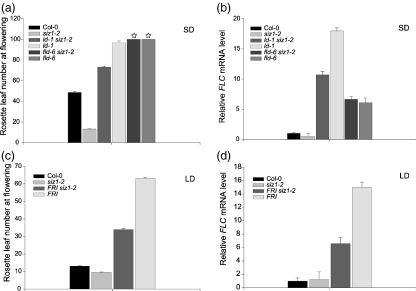
*siz1* partially suppresses the late flowering of *FRI* and *ld*-*1*, but not of *fld*-*6*. (a) The flowering times of wild-type (Col-0), *siz1*-*2*, *ld*-*1 siz1*-*2*, *ld*-*1*, *fld*-*6 siz1*-*2* and *fld*-*6* plants were analyzed under short days (SD). Stars indicate that flowering had not occurred after producing more than 100 rosette leaves in *fld*-*6* and *fld*-*6 siz1*-*2* plants. (b) Relative *FLC* mRNA levels were determined in 14-day-old SD-grown wild-type (Col-0), *siz1*-*2*, *ld*-*1 siz1*-*2*, *ld*-*1*, *fld*-*6 siz1*-*2* and *fld*-*6* seedlings by quantitative PCR. (c) Flowering times of wild-type (Col-0), *siz1*-*2*, *FRI siz1*-*2* and *FRI* plants were estimated under long days (LD). (d) Relative *FLC* mRNA levels in wild-type (Col-0), *siz1*-*2*, *FRI siz1*-*2* and *FRI* seedlings were determined by quantitative PCR. RNA was isolated from 10-day-old seedlings grown under long days (LD). Data illustrated in (a) and (c) are means ± SE of 15–20 plants per analysis.

To determine if *SIZ1* regulates floral repressors, the expression of *FLC*, *FLOWERING LOCUS M* (*FLM*)/*MADS AFFECTING FLOWERING1* (*MAF1)*, *MAF2*, *MAF3*, *MAF4* and *MAF5*, and *SHORT VEGETATIVE PHASE* (*SVP*) were analyzed (Hartmann *et al.*, 2000; Ratcliffe *et al.*, 2001, 2003; Scortecci *et al.*, 2001). *FLC* transcript abundance in *siz1* seedlings was slightly reduced compared with wild-type seedlings under SD (Figure 5b). *MAF4* mRNA abundance was substantially lower in *siz1* plants compared with wild type under both LD and SD, whereas, expression of the other floral repressors *FLM/MAF1*, *SVP*, *MAF2*, *MAF3* and *MAF5* was not affected in *siz1* plants. Together, these results indicate that *SIZ1* positively regulates *FLC* and *MAF4* gene expression (Figures 4a and 5b).

To test whether reduced *FLC* expression levels contribute to early flowering of *siz1*, a double mutant was made containing *siz1*-*2* and an *FLC* null allele (*flc*-*3*) (Michaels and Amasino, 1999). Under LD, *flc*-*3* flowered slightly earlier than wild-type plants (Figure 4b). The flowering time of *flc*-*3 siz1*-*2* was earlier than that of *flc*-*3* or *siz1*-*2* plants (Figure 4b). Consistent with a previous report (Michaels and Amasino, 2001), the flowering time of *flc*-*3* was slightly earlier than in wild-type plants under SD (Figure 4b). Double-mutant *flc*-*3 siz1*-*2* plants flowered at the same time as *siz1*-*2* plants, but flowered much later than *flc*-*3* plants under SD (Figure 4b). These results indicate that reduced *FLC* expression does not contribute to the early flowering of *siz1* plants. Thus, in the Columbia background, *SIZ1* regulates the transition to flowering mainly through an *FLC*-independent pathway(s).

*SOC1* transcript levels were greater in *siz1* compared with wild-type plants under both LD and SD (Figure 4a). Flowering time of *soc1*-*2 siz1*-*2* was similar to that of *soc1*-*2* plants under LD, suggesting that the early flowering of *siz1* under LD is mainly dependent on elevated *SOC1* expression levels (Figure 4c). The flowering time of *soc1*-*2 siz1*-*2* was intermediate to that of *siz1*-*2* and *soc1*-*2* under SD. It appears that the early flowering of *siz1* under SD was only partially dependent on elevated *SOC1* expression (Figure 4c).

### The interaction of SIZ1 with FRI and the autonomous pathway genes

Although reduction of *FLC* transcript abundance by the *siz1* mutation does not affect flowering time in the Columbia background (i.e. *fri* null, basal *FLC* expression), *SIZ1* appears to be required for full *FLC* expression in late-flowering autonomous pathway mutants and a *FRI* background. Double mutants were made between *siz1*-*2* and dysfunctional alleles of late-flowering autonomous pathway mutants, such as *luminidependens* (*ld*-*1*) and *fld*-*6* (Lee *et al.*, 1994b; Sanda and Amasino, 1996). Also, an active *FRI* allele was introduced into *siz1*-*2* plants by crossing with *FRI*-Col (Michaels and Amasino, 1999; Sheldon *et al.*, 1999). As established in previous studies (Lee *et al.*, 1994b; Michaels and Amasino, 1999), *ld-1* and *FRI*-Col plants flowered substantially later than Col-0 or *siz1*-*2* plants, which results from greater *FLC* transcript abundance relative to wild-type plants (Figure 5). The *siz1*-*2* mutation partially suppressed the late-flowering phenotype of *ld*-*1* and *FRI*-Col plants (Figure 5a,c). Consistent with the flowering time phenotype, *FLC* expression levels were reduced in *ld*-*1 siz1*-*2* and *FRI* *siz1*-*2* compared with that of the late-flowering parental plants *ld*-*1* and *FRI*-Col, respectively (Figure 5b,d). Note that exogenous SA treatment does not accelerate the flowering time of autonomous pathway mutants (Martinez *et al.*, 2004). Together, these results suggest that *SIZ1* activates *FLC* expression, possibly through an SA-independent pathway, which is required for full *FLC* activation by *ld*-*1* and *FRI*. However, interestingly, *fld*-*6* and *fld*-*6 siz1*-*2* plants did not flower after producing more than 100 rosette leaves under SD (Figure 5a). Moreover, increased *FLC* expression in *fld*-*6* was not suppressed by the *siz1* mutation (Figure 5b). These results suggest that FLD may be required for *SIZ1* to promote *FLC* expression. No difference in *FLD* transcript abundance was observed between wild-type and *siz1* plants (data not shown), indicating that *SIZ1* does not regulate expression of *FLD*.

### SIZ1 facilitates SUMO1 modification of FLD

**Figure6:**
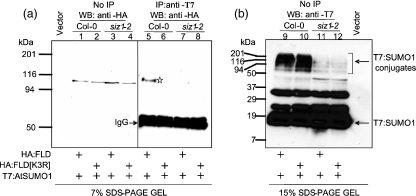
*SIZ1* mediates SUMO modification of FLD. (a) HA:FLD or HA:FLDK3R (K287R, K693R and K770R) and T7:AtSUMO1 translational fusions were co-expressed in wild-type (Col-0) or *siz1*-*2* protoplasts (Jin *et al.*, 2001). T7:AtSUMO1 and HA:FLD or HA:FLDK3R were co-immunoprecipitated (IP) from extracts, and were then detected on the western blot (WB) with anti-HA. No IP is protoplast lysate before IP. The star indicates the position of sumoylated FLD proteins (right panel). HA:FLD and HA:FLDK3R transient expression levels were similar in *siz1* and wild-type (Col-0) plants (left, No IP panel); vector, total protein extract from protoplasts transformed with the empty vector. (b) A WB with anti-T7 was used to determine the expression level of T7:AtSUMO1 in the No-IP samples from (a). Free T7:AtSUMO1 transient expression levels were similar in *siz1* and wild-type (Col-0) plants (lower arrow). T7:AtSUMO1 conjugates (upper arrow) were nearly undetectable in *siz1*-*2* (lanes 11 and 12) compared with wild-type (Col-0) (lanes 9 and 10) protoplasts. No IP and vector are as in (a).

Three potential sumoylation motifs (IK287VE, PK693AD and IK770AE) in FLD were identified by SUMOplot (http://www.abgent.com/tool/sumoplot) analyses. Thus, FLD could be a potential SUMO target protein (Schmidt and Muller, 2003). To test this possibility, human influenza hemagglutinin (HA)-tagged FLD (HA:FLD) was transiently expressed in wild-type or *siz1*-*2* protoplasts (Jin *et al.*, 2001). Proteins from wild-type and *siz1*-*2* protoplasts were separated by SDS-PAGE gel (Figure 6a, left panel). Interestingly, a single anti-HA reactive protein was detected after expressing HA:FLD in wild-type protoplasts, even though two anti-HA reactive proteins bands are predicted (sumoylated and unsumoylated) (Figure 6a, lane 1). Presumably SUMO modification of FLD *in planta* is very efficient, and only the sumoylated protein is observed. The molecular mass of AtSUMO1 is 12 kDa. Thus, it is expected that sumoylated FLD should have a molecular mass that is 12 kDa larger than unsumoylated FLD. However, the HA:FLD band resulting from wild type (Figure 6a, lane 1) appeared to be only 3.5–4 kDa larger than the corresponding band from *siz1*-*2* protoplasts (Figure 6a, lane 3). There are probably two possibilities that cause this aberrant protein mobility on the SDS-PAGE. First, SUMO modification of proteins induces conformational change in some proteins (Steinacher and Schar, 2005). The sumoylated FLD may undergo a conformational change that may be insensitive to SDS treatment. Second, sumoylation and other post-translational modification processes (e.g. phosphorylation) that coordinately regulate protein function, etc. are often interdependent (Vu *et al.*, 2007). Perhaps the smaller molecular mass differences between sumoylated and unsumoylated FLD are the consequence of additional post-translational processes.

To determine if the slightly larger molecular mass of the protein in wild type is indeed the result of sumoylation, HA:FLD and T7:AtSUMO1 were transiently co-expressed in wild-type and *siz1*-*2* protoplasts (Jin *et al.*, 2001). Total protein was isolated from protoplasts under denaturing conditions to minimize desumoylation of the conjugated peptides by SUMO proteases during extraction. Protoplast lysates were then diluted with immunoprecipitation buffer, and T7:AtSUMO1 was immunoprecipitated with anti-T7. A single peptide band was detected with anti-HA from immunoprecipitated proteins of wild-type protoplasts, indicating that HA:FLD physically interacts with T7:AtSUMO1 (Figure 6a, lane 5). However, the anti-HA reactive band was almost non-detectable on the immunoblot of proteins from *siz1* protoplasts (Figure 6a, lane 7). These results indicate that SIZ1 facilitates SUMO1 conjugation to FLD.

K to R mutations in sumoylation motifs block SUMO conjugation to protein substrates (Hilgarth *et al.*, 2004; Miura *et al.*, 2005). Consequently, K residues in the three predicted sumoylation motifs of FLD were substituted with R residues (HA:FLDK3R), and Co-IP analysis was performed as described above (Figure 6a). T7:AtSUMO1 conjugation to HA:FLDK3R was not detected on the immunoblot of protein isolated from wild-type or *siz1*-*2* protoplasts (Figure 6a, compare lanes 6 and 8). These results indicate that the K residues in one or more of the three sumoylation motifs are necessary for SUMO1 modification of FLD, and that the capacity for AtSUMO1 conjugation to FLD is impaired in *siz1*.

### SIZ1-mediated SUMO modification of FLD represses H4 deacetylation of FLC chromatin

**Figure7:**
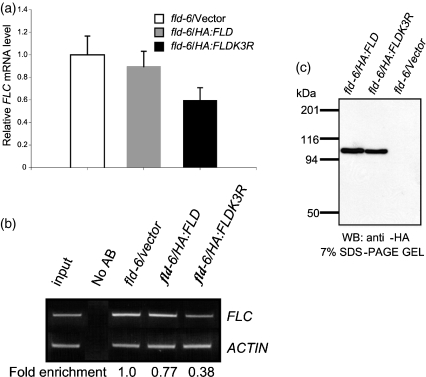
SIZ1-mediated SUMO modification of FLD represses deacetylation of histone H4 in *FLC* chromatin. (a) Relative *FLC* mRNA levels were determined by quantitative PCR in *fld*-*6* protoplasts expressing vector, *HA:FLD* or *HA:FLDK3R*. Data are means ± SD (*n* = 4). (b) The acetylation state of H4 in *FLC* chromatin was assessed by chromatin immunoprecipitation (ChIP) analysis in *fld*-*6* protoplasts expressing FLD or FLDK3R. Input is *fld*-*6* chromatin before immunoprecipitation that was isolated from protoplasts transformed with empty vector; No AB and ACTIN are as in (b). The fold enrichment in H4 acetylation of *fld*-*6* protoplasts expressing FLD or FLDK3R overexpressing the empty vector is shown. (c) HA:FLD and HA:FLDK3R were expressed equally in *fld*-*6* protoplasts in this experiment.

Effects of FLD and FLDK3R (constitutively unsumoylated) on *FLC* expression were evaluated to determine if SIZ1-mediated sumoylation of FLD alters its activity (Figure 7a). *HA:FLD*, *HA:FLDK3R* or empty vector were transiently expressed in *fld*-*6* protoplasts that were isolated from pre-flowering plants grown under SD. After a 40-h incubation, protoplasts were harvested and the *FLC* mRNA level was determined by quantitative real-time PCR (Figure 7a). Transient, but equivalent, expression in protoplasts of *HA:FLDK3R* (unsumoylated) reduced *FLC* expression to a greater extent than expression of HA:FLD (sumoylated) relative to vector control (Figure 7a,c).

*FLD* mutations, which disturb protein function, cause *FLC* transcript accumulation and late flowering that is linked to hyperacetylation of histone 4 (H4) in chromatin associated with the first intron of *FLC* (He *et al.*, 2003). Consequently, the H4 acetylation status of *FLC* chromatin in the protoplast samples used in the experiments presented in Figure 7(a) were evaluated using a chromatin immunoprecipitation (ChIP) assay (Figure 7b). Consistent with the *FLC* mRNA level seen in Figure 7(a), H4 in *FLC* chromatin was less acetylated in protoplasts expressing *HA:FLDK3R* compared with those expressing *HA:FLD* (Figure 7b). These results suggest that SIZ1-mediated SUMO modification of FLD inhibits its ability to repress *FLC* expression by reducing acetylation of H4 in *FLC* chromatin. This SUMO-mediated regulatory mechanism appears to be required for full *FLC* activation by *FRI*.

## Discussion

### Mechanisms for SIZ1 regulation of flowering in the Columbia background

Under LD, the flowering time of *soc1*-*2 siz1*-*2* plants was simililar to that of *soc1*-*2*, suggesting that the slightly earlier flowering of *siz1* under LD is mainly the result of elevated *SOC1* expression (Figure 4). The photoperiodic pathway activates *SOC1* expression through *FT*, which is repressed by *FLC* (Searle *et al.*, 2006; Wigge *et al.*, 2005). However, expression of *FT* and *FLC* was nearly unaffected by *siz1* under LD (data not shown and Figure 5d, respectively). Thus, increased *SOC1* expression in *siz1* plants is not the result of elevated expression of *FT* or of reduced expression of *FLC*. If the activation of *SOC1* expression is the result of increased SA levels caused by *siz1*, then the flowering time of *nahG* *siz1*-*2* plants should be similar to that of *nahG* plants under LD. However, the flowering time of *nahG* *siz1*-*2* plants was similar to that of *siz1*-*2* under LD (Figure 3b), suggesting that SA does not activate *SOC1* expression, which is consistent with a previous report that the UV-C light-induced accumulation of SA does not affect *SOC1* expression (Martinez *et al.*, 2004). Therefore, *SIZ1* represses *SOC1* expression through *FT*- and *FLC*-independent pathways, and through an unknown SA-independent pathway. The *siz1* mutation substaintially reduced the expression of the floral repressor *MAF4*, which may also contribute to early flowering of *siz1* under LD.

Under SD, the substaintial early flowering phenotype of *siz1* is mainly the result of elevated SA levels (i.e. the flowering time of *nahG* *siz1*-*2* was similar to that of *nahG* plants). Under SD, *SIZ1* function on flowering time also showed little dependence on *FLC* (i.e. the flowering time of *flc*-*3 siz1*-*2* was similar to that of *siz1*-*2* plants), indicating that under SD, SA promotes transition to flowering mainly through *FLC*-independent pathway(s). These results further indicate that SA accelerates flowering through pathways that are independent of the vernalization and the autonomous pathways, as these two pathways promote transition to flowering through repression of *FLC* expression. SA could facilitate transition to flowering by shortening the circadian period (Mizoguchi *et al.*, 2002). However, rhythmic expression of *CCA1* and *CCR2* were not altered in *siz1* plants (Figure S1), suggesting that SA does not regulate the circadian clock. Moreover, GA-dependent floral promotion is operative in *siz1* plants that contain a high level of SA (Figure S2). Thus, it is likely that SA accelerates flowering through pathways that are independent of photoperiodic- and vernalization-dependent pathways, and the autonomous and GA-dependent pathways. Despite the major role of SA in the early flowering of *siz1* under SD, the flowering time of *nahG* *siz1*-*2* was slightly earlier than that of *nahG* plants under SD (Figure 3b), indicating that *siz1* also accelerates flowering through SA-independent machanisms under SD. This may include activation of *SOC1* and/or repression of *MAF4* expression. Although *SIZ1* is now strongly implicated in SA accumulation, and in its subsequent affect on flowering time under SD, the sumoylation targets of SIZ1 that affect SA accumulation remain to be discovered. SA-mediated flowering control has escaped major attention, perhaps because it is dependent on the interaction of particular environmental and genetic background conditions. However, the other major flowering signal pathways are also similarly affected, and the roles of SA and sumoylation in flowering time control may prove as important as other major signal systems.

### Possible mechanism for *FLC* activation by *SIZ1*

Interestingly, activated *FLC* expression in *fld*-*6* was not affected by *siz1*, wheareas *siz1* caused partial suppression of *FLC* expression in *FRI* and *ld*-*1* plants. The first interpretation of these results is that *SIZ1* promotes *FLC* expression by inhibiting FLD activity, which is required for the full activation of *FLC* expression in *FRI* and *ld*-*1* plants. Alternatively, *SIZ1* and *FLD* may function in independent pathways, and activation of *FLC* expression by *fld*-*6* could overcome repression of *FLC* expression caused by *siz1*. However, we also found that SIZ1 facilitates sumoylation of FLD, which represses FLD activity. This strongly supports the first interpretation. In addition, partial suppression of *FLC* expression in *FRI* and *ld*-*1* plants by *siz1* is difficult to explain by the alternative interpretation, as *FRI* or *ld*-*1* also causes strong *FLC* activation. Therefore, it is likely that the partial suppression of *FLC* expression in an *FRI* and *ld*-*1* background by *siz1* mutation is, at least in part, caused by the inhibition of FLD activity by SIZ1-mediated SUMO modification. However, we must consider the possibility that *SIZ1* also activates *FLC* expression through an FLD-independent mechanism.

### Possible mechanisms by which SUMO modification of FLD affects HDAC activity

We have found that sumoylation/desumoylation of FLD controls histone acetylation/deacetylation, but the mechanism(s) by which SUMO modification of FLD affects HDAC activity remains unelucidated. The FLD homolog, KIAA0601/LSD1, has lysine-specific demethylase activity that is associated with numerous co-repressor complexes, such as CoREST, BHC80 and HDAC in humans (Humphrey *et al.*, 2001; Shi *et al.*, 2003, 2004). LSD1 and HDAC1 function cooperatively in a co-repressor complex (Lee *et al.*, 2006). CoREST induces LSD1 demethylation activity, but BHC80 negatively regulates LSD1 demethylation activity (Shi *et al.*, 2005). Interestingly, LSD1 undergoes some unknown post-translational modification (You *et al.*, 2001). LSD1 contains three potential sumoylation sites, which are likely targets for SUMO modification. Although the biochemical function of FLD remains unknown, it is possible that the activity and regulatory mechanisms of FLD are similar to that of LSD1. It is possible that SUMO modification of FLD enhances and/or inhibits interaction with an FLD repressor and/or activator, respectively, and consequently inhibits HDAC function in the repressor complex. Identification and characterization of FLD interacting partners will help us to understand mechanisms by which AtSIZ1-mediated sumoylation affects FLD activity, and that understanding will have broad scientific relevance to both plant and animal systems.

## Experimental procedures

### Plant materials

Genotypes used in all experiments were in the Columbia genetic background. The *siz1*-*2* (SALK_065397) and *siz1*-*3* (SALK_034008) lines were obtained from ABRC at Ohio State University (http://www.biosci.ohio-state.edu/pcmb/Facilities/abrc/abrchome.htm; Miura *et al.*, 2005). Early and late flowering mutants, *flc*-*3*, *ft*-*1*, *soc1*-*2*, *gi*-*2*, *co*-*1*, *ld*-*1* and *FRI*-SF2 in the Columbia background (*FRI*-Col) were described previously (Fowler *et al.*, 1999; Kardailsky *et al.*, 1999; Kobayashi *et al.*, 1999; Koornneef *et al.*, 1991; Lee *et al.*, 1994b, 2000; Michaels and Amasino, 1999). *fld*-*6* (SAIL_642 C05) was isolated from T-DNA mutant SAIL lines, which were kindly provided by Dr R.M. Amasino (University of Wisconsin, http://www.wisc.edu). Homozygous double mutants were obtained by crossing various flowering-time mutants with *siz1*-*2*. The presence of *siz1*-*2* and *fld*-*6* mutations were analyzed by diagnostic PCR analysis according to the SALK T-DNA verification protocol (http://signal.salk.edu), and the presence of the *FRI*-SF2, *ld*-*1*, *flc*-*3*, *gi*-*2*, *co*-*1*, *ft*-*1* and *soc1*-*2* mutations was analyzed according to a previous report (Lee *et al.*, 2000; Moon *et al.*, 2005).

### Growth conditions

To break seed dormancy, seeds were stratified on soil for 4 days at 4°C before transfer to normal growth conditions. Plants were grown at 23°C in a greenhouse under LD (16-h light/8-h dark), whereas for SD (8-h light/16-h dark), plants were grown at 22°C under fluorescent lights (100 μmol m^−2^ sec^−1^) in growth chambers, which were equipped with seven ALTO PLUS T8 fluorescent lamps (#F32T8/TL735/PLUS/ALTO; Philips, http://www.philips.com) and one PLANT & AQUARIUM 40W lamp (GE; http://www.ge.com). For exogenous GA treatment, 100 μM GA_3_ was sprayed twice onto 7- or 14-day-old seedlings grown under either LD or SD. The flowering time is estimated based on the number of rosette leaves formed by the primary shoot apical meristem prior to flowering under LD and SD, as described above. At least 15–20 plants were used to determine the flowering time of each genotype.

### RT-PCR

Total RNA was isolated using the PureLink Micro-to-Midi Total RNA Purification System (*#*12183-018; Invitrogen, http://www.invitrogen.com) according to the manufacturer’s protocol. A 200-ng sample of RNA was used as the template for first-strand cDNA synthesis with the ThermoScript RT-PCR System (*#*11146-016; Invitrogen) and an oligo (dT_21_) primer. Specific gene expression levels were analyzed by semiquantitative RT-PCR or real-time PCR (Miura *et al.*, 2005). Primer sequences for gene amplifications are listed in Table S2.

### In vivo sumoylation assay

*In vivo* sumoylation was assayed as described previously by Hilgarth *et al.* (2004). HA:FLD or HA:FLDK3R was transiently co-expressed with T7:AtSUMO1 in protoplasts prepared from 14-day-old wild-type and *siz1*-*2* seedlings by polyethylene glycol-mediated transformation (Jin *et al.*, 2001). After 40-h incubation, immunoprecipitation was performed using T7 tag monoclonal antibody (#69522-3; Novagen, http://www.emdbiosciences.com) with protein-A-sepharose CL-4B (#17-0963-03; Amersham, http://www.amersham.com). Immunoprecipitated proteins were released in 2× SDS sample buffer, separated by SDS- PAGE, and detected by western blotting using anti-HA monoclonal antibodies (#sc-7392; Santa Cruz Biotechnology, Inc., http://www.scbt.com) (Jin *et al.*, 2001).

### Chromatin immunoprecipitation analysis

The chromatin immunoprecipitation experiments were performed as described previously (He *et al.*, 2003). The primer pair CH1/H12 and JP1595/JP1596 were used to amplify the first intron region of *FLC* chromatin and ACTIN 2/7, respectively (see Table S2 for primer sequence) (He *et al.*, 2003). SD-grown 20-day-old wild-type, *siz1*-*2* and *fld*-*6* plants and anti-acetylated H4 antibody were used for ChIP analyses. To check the activity of the sumoylation-deficient mutant, FLDK3R, HA:FLD or HA:FLDK3R were transiently expressed in SD-grown 20-day-old *fld*-*6* protoplasts. After 40 h of incubation, ChIP analysis was performed to determine the acetylation status of H4 in *FLC* chromatin as described above. The fold enrichment in H4 acetylation was calculated as follows: *FLC* was first normalized to *ACTIN* in each sample, and these values were normalized against their respective wild type or vector controls.

### Rhythm analysis

Rhythm analysis was performed as described by Kim *et al.* (2005).

## Supplementary material

The following supplementary material is available for this article online:

Figure S1 Lesions in SIZ1 do not impair clock-controlled gene expression.

Figure S2 Exogenous gibberellin (GA) treatment accelerates flowering in both wild-type and siz1 plants.

Table S1 Period of circadian rhythms estimation in different light conditions at 21°C.

Table S2 Primers for RT-PCR, real-time PCR or chromatin immunoprecipitation (ChIP) analysis.

Table S3 Primers for subcloning.

Appendix S1 Experimental procedures: plasmid construction.

This material is available as part of the online article from http://www.blackwell-synergy.com
