# Supplementary figures and images for: The SUMO E3 ligase, AtSIZ1, regulates flowering by controlling a salicylic acid-mediated floral promotion pathway and through affects on FLC chromatin structure
Source: Plant J. 2008 Feb;53(3):530–40. doi: 10.1111/j.1365-313X.2007.03359.x (PMC2254019; doi:10.1111/j.1365-313X.2007.03359.x)

**
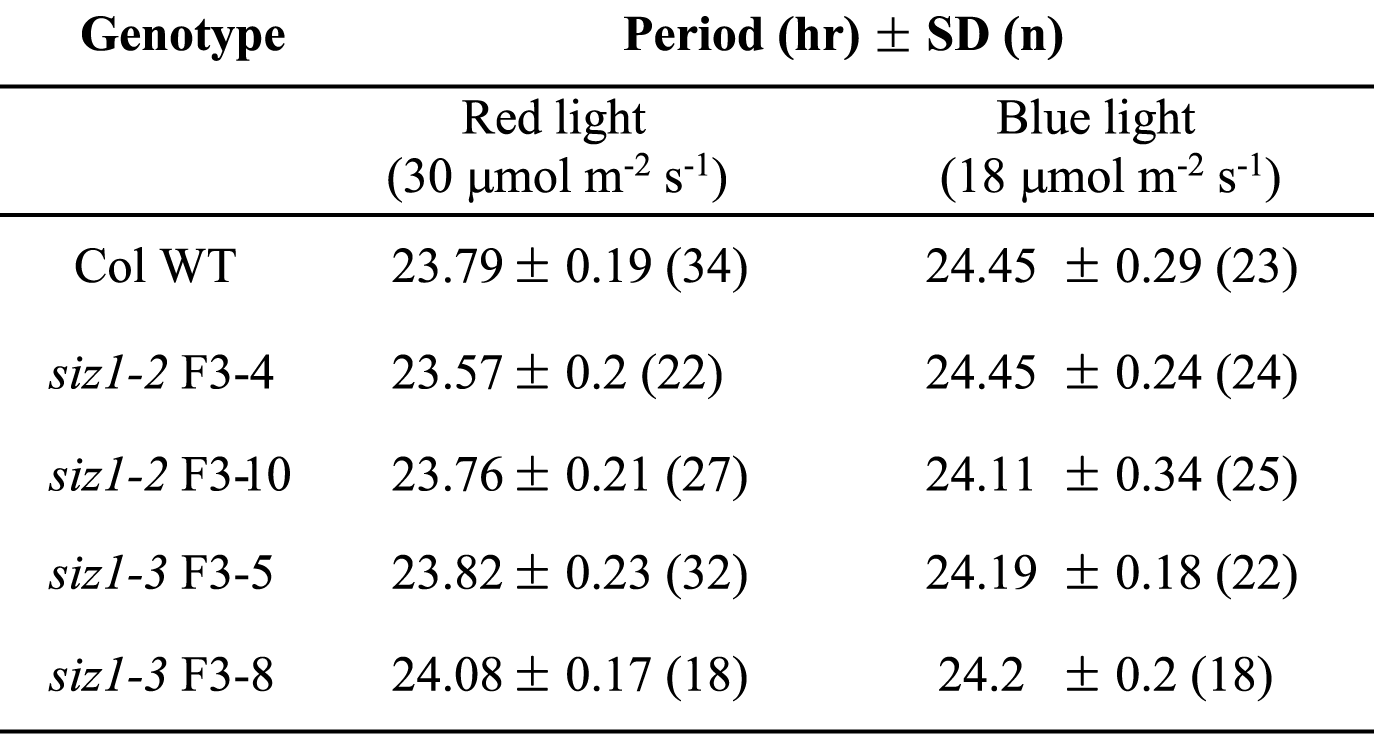
**

Supplement: Table S1 — Period of circadian rhythms estimation in different light conditions at 21°C. [file tpj0053-0530-sm-table1.doc]
