# Supplementary material for: The SUMO E3 ligase, AtSIZ1, regulates flowering by controlling a salicylic acid-mediated floral promotion pathway and through affects on FLC chromatin structure
Source: Plant J. 2008 Feb;53(3):530–40. doi: 10.1111/j.1365-313X.2007.03359.x (PMC2254019; doi:10.1111/j.1365-313X.2007.03359.x)
Supplement: Table S2 — Primers for RT-PCR, real-time PCR or chromatin immunoprecipitation (ChIP) analysis. [file tpj0053-0530-sm-table2.doc]

| Primer name | Primer Sequences |
| --- | --- |
| FT5-3 | 5'-cctgctacaactggaacaaccttt-3' |
| FT3-2 | 5'-ataggcatcatcaccgttcgttactcg-3' |
| SOC1-1 | 5'-gtttctgaagaaaatatgcagcatt-3' |
| SOC1-2 | 5'-gaacaaggtaacccaatgaacaa-3' |
| CCA1-5' | 5'-cgtgtggctcaaacactccg-3' |
| CCA1-3' | 5'-tcctgtgtttctcttctcct-3' |
| FLC-5' | 5'-atgggaagaaaaaaactagaa-3' |
| FLC-3' | 5'-ctaattaagtagtgggag-3' |
| FLM-RT-5' | 5'-gtagagctaggaaggcagaactga-3' |
| FLM-RT-3' | 5'-ccgaaggaggtacaacactgatcc-3' |
| MAF2-RT-5' | 5'-acattgtgggtctccggtgattaggatc-3' |
| MAF2-RT-3' | 5'-aatcaggctgtaagtttaaggtgaaagc-3' |
| MAF3-5'RT | 5'-GAAGAAAAAAAGCAAACACATTTTGGGTCC-3' |
| MAF3-3'RT | 5'-AAGAACTCTGATATTTGTCTACTAAGGTAC-3' |
| MAF4-5'RT | 5'-ATTAGGTCAGAAGAATTAGTCGGAGAAAAC-3' |
| MAF4-3'RT | 5'-CTTGGATGACTTTTCCGTAGCAGGGGGAAG-3' |
| MAF5-5'RT | 5'-GGGGATTAGATGTGTCGGAAGAGTGAAG-3' |
| MAF5-3'RT | 5'-GATCCTGTCTTCCAAGGTAACACAAAGG-3' |
| SVP-5' | 5'-cgctctcatcatcttctcttccac-3' |
| SVP-3' | 5'-gctcgttctcttccgttagttgc-3' |
| FLC-5R1 (real-time PCR) | 5'- cactatgagctacttgaacttgtgg-3' |
| FLC-3R1 (real-time PCR) | 5'- tggctctagtcacggagagg-3' |
| TUBULIN-5' | 5'-ctggaaaatcgaatgctgaagac-3' |
| TUBULIN-3' | 5'-aagttccatctgacgtatgtcaa-3' |
| CH1 | 5'-ctgcgaccatgatagatacatgaga-3' |
| H12 | 5'-ttcactcaacaacatcgagcacg-3' |
| JP1595 | 5'-cgtttcgctttccttagtgttagct-3' |
| JP1596 | 5'-agcgaacggatctagagactcaccttg-3' |
