# Supplementary material for: The SUMO E3 ligase, AtSIZ1, regulates flowering by controlling a salicylic acid-mediated floral promotion pathway and through affects on FLC chromatin structure
Source: Plant J. 2008 Feb;53(3):530–40. doi: 10.1111/j.1365-313X.2007.03359.x (PMC2254019; doi:10.1111/j.1365-313X.2007.03359.x)
Supplement: Table S3 — Primers for subcloning. [file tpj0053-0530-sm-table3.doc]

| Primer name | Primer Sequences |
| --- | --- |
| AtSIZ1-5'-XmaI | 5'-cccgggatggatttggaagctaattg-3' |
| AtSIZ1-3'-SpeI | 5'-actagtctcagaatccgagtcaat-3' |
| Psiz1-full-5'XmaI | 5'-cccgggttcttcaacatccacagagag-3' |
| Psiz1-3'-XmaI | 5'-cccgggcatgtcttcaacaccagaca-3' |
| Psiz1-3'BamHI | 5'-ggatccaccatgtcttcaacaccagaca-3' |
| FLD-5'-XmaI | 5'-cccgggaaatggtctcattctccgcacc-3' |
| FLD-3'-Acc65I | 5'-ggtaccctaaccagattttgtgcctga-3' |
| FLDK287R-R | 5'-acctcaacccttatatccacatctggatc-3' |
| FLDK287R-F | 5'-ggatataagggttgaggtagcgtttaa-3' |
| FLDK693R-R | 5'-gatctgctcttgggtcctcgtttctc-3' |
| FLDK693R-F | 5'-ggacccaagagcagatcagcactcaaa-3' |
| FLDK770R-R | 5'-gctcggctctaatagaagcaatgactga-3' |
| FLDK770R-F | 5'-ttctattagagccgagcgaactggtc-3' |
| AtSUMOI-5'-BamHI | 5'-ggatccatatgtctgcaaaccaggagg-3' |
| AtSUMO1-3'-XmaI | 5'-cccgggttagccaccagtctgatgg-3' |
