# Supplementary material for: The SUMO E3 ligase, AtSIZ1, regulates flowering by controlling a salicylic acid-mediated floral promotion pathway and through affects on FLC chromatin structure
Source: Plant J. 2008 Feb;53(3):530–40. doi: 10.1111/j.1365-313X.2007.03359.x (PMC2254019; doi:10.1111/j.1365-313X.2007.03359.x)
Supplement: Appendix S1 — Experimental procedures: plasmid construction. [file tpj0053-0530-sm-appendixs1.doc]

**Plasmid construction**

To generate *Pro_SIZ1_:SIZ1:GFP*, the full-length *SIZ1* cDNA without the termination codon was amplified with gene specific primers (AtSIZ1-5'-XmaI and AtSIZ1-3'-SpeI) and the *SIZ1* promoter was amplified from genomic DNA (Psiz1-full-5'XmaI and Psiz1-3'-XmaI). *SIZ1* cDNA was inserted in frame at the XmaI and SpeI sites of *pCambia1302* (*pCambia1302-SIZ1:GFP*). The *SIZ1* promoter was inserted at the XmaI site of *pCambia1302-SIZ1:GFP*. To generate *Pro_SIZ1_:*GUS:GFP, the *SIZ1* promoter was amplified with gene specific primers (Psiz1-full-5'XmaI and Psiz1-3'BamHI) and digested with the XmaI and BamHI. Digested products were inserted into XmaI and BglII sites of *pCambia1303*. To add the Hemagglutinin (HA) epitope at the N terminus of FLD, the FLD coding region was amplified with gene specific primers (FLD-5'-XmaI and FLD-3'-Acc65I) and then ligated in-frame to the C terminus of the HA epitope. *FLDK3R* was generated by PCR using the primers: FLDK287R-R, FLDK287R-F, FLDK693R-R, FLDK693R-F, FLDK770R-R and FLDK770R-F. To generate *T7:AtSUMO1*, *AtSUMO1* cDNA was generated by PCR using primers: AtSUMOI-5'-BamHI and AtSUMO1-3'-XmaI, which was then fused in-frame to the C terminus of the T7 epitope. *HA:FLD*, *HA:FLDK3R* and *T7:AtSUMO1* were constructed into a CaMV 35S promoter controlled transient expression vector (Jin et al., 2001). Primer sequences are listed in supplement table 3.
