# Supplementary material for: The SUMO E3 ligase, AtSIZ1, regulates flowering by controlling a salicylic acid-mediated floral promotion pathway and through affects on FLC chromatin structure
Source: Plant J. 2008 Feb;53(3):530–40. doi: 10.1111/j.1365-313X.2007.03359.x (PMC2254019; doi:10.1111/j.1365-313X.2007.03359.x)
Supplement: Supplementary file 7 [file tpj0053-0530-sm-legends.doc]

**Supplementary figure 1.** Lesions in *SIZ1* do not impair clock-controlled gene expression. (A) *CCA1* mRNA abundances in SD grown 14-d-old wild-type (Col-0) and *siz1-2* seedlings were determined by using RT-PCR analysis for every 4 h under SD. The graph shows the mean level of *CCA1* expression relative to the *TUBULIN* control. White and black box illustrate periods of light and dark, respectively. (B) *CCR2::LUC* expression in wild-type (WT), *siz1-2* (F3-4 and 10) and *siz1-3* (F3-5 and 8) seedlings. Plants were entrained in 12-h-white light /12-h-dark cycles and transferred to constant red light (RR; 30 μmol m^-2^ s^-1^), and bioluminescence was monitored every 2 h for 6 d. Traces represent averages from at least 20 seedlings from two independent experiments. Period estimates are shown in supplementary table 1.

**Supplementary figure 2.** Exogenous GA treatment accelerates flowering in both wild type and *siz1* plants. Number of rosette leaves at flowering of plants was estimated under LD or SD. Illustrated are data from plants without (-GA) or after 100 μM GA_3_ (+GA) treatment. Data are means ± SE, of 15-20 plants per treatment.

**Supplementary table 1.** Period estimation in different light conditions at 21℃.

Plants were entrained as described in Supplementary figure 1B and transferred to indicate light conditions for period determination. Period estimates (variance-weighted mean ± variance-weighted SD) were obtained as described (Millar, A.J., Straume, M., Chory, J., Chua, N.-H., Kay, S.A. (1995) The regulation of circadian period by phototransduction pathways in Arabidopsis. *Science* **267:** 1163–1166)

**Supplementary table 2.** Primers for RT-PCR, real-time PCR or ChIP analysis

**Supplementary table 3.** Primers for sub-cloning
